# MRI biomarkers of primary CNS lymphoma outcome after MATRix: real world performance

**DOI:** 10.3389/fonc.2026.1769381

**Published:** 2026-06-25

**Authors:** Nitin Menon, Isabelle Naylor, Emmanuel Buba, Alberto Schena, Paul S. Morgan, Christopher P. Fox, Stefanie Thust

**Affiliations:** 1 Neuroradiology Department, Queen’s Medical Centre, Nottingham University Hospitals NHS Trust, Nottingham, United Kingdom; 2 School of Medicine, University of Nottingham, Nottingham, United Kingdom; 3 Department of Haematology, Nottingham University Hospital NHS Trust, Nottingham, United Kingdom; 4 Radiological Sciences, Mental Health and Neurosciences, School of Medicine, University of Nottingham, Nottingham, United Kingdom; 5 NIHR Nottingham Biomedical Research Centre, Nottingham University Hospitals NHS Trust, Nottingham, United Kingdom

**Keywords:** lymphoma - diagnosis, lymphoma prognosis, PCNSL = primary central nervous system lymphoma, PCNSL imaging, response assessment

## Abstract

**Introduction:**

Despite treatment advances, accurate prognostication for patients with newly diagnosed primary central nervous system lymphoma (PCNSL) remains unsatisfactory. This retrospective cohort study evaluated whether T1 contrast-enhanced (T1CE) tumour volume and apparent diffusion coefficient (ADC) metrics on magnetic resonance imaging (MRI) predict survival in PCNSL patients treated uniformly with MATRix chemotherapy.

**Methods:**

Sixty-five patients underwent pre-treatment MRI with volumetric and diffusion analysis. T1CE tumour volume, ADCmean, ADCmin and normalised diffusion parameters were assessed. Survival outcomes were analysed using univariate Cox regression for overall survival (OS) and progression-free survival (PFS), including disease-specific endpoints.

**Results:**

None of the tested imaging variables were significantly associated with OS or PFS, including T1CE tumour volume and ADC measures. Baseline T1CE and diffusion biomarkers did not predict survival in MATRix-treated PCNSL.

**Discussion:**

These results highlight limitations of standard MRI techniques for prognostication underscoring the need for integrated multiparametric models incorporating imaging and biological data to improve outcome prediction.

## Introduction

Primary central nervous system lymphoma (PCNSL) is a rare, aggressive extranodal non−Hodgkin lymphoma, representing approximately 4% of all central nervous system (CNS) tumours. In almost all cases, PCNSL corresponds histologically to diffuse large B−cell lymphoma (DLBCL), and its protection within the blood-brain-barrier poses significant challenges for treatment delivery and response assessment (1).

High-dose methotrexate-based regimens are the foundation of PCNSL chemotherapy protocols. The MATRix protocol (methotrexate, cytarabine, thiotepa, rituximab) is established as first-line remission-induction for fit, immunocompetent patients under 70 years (2, 3), followed by autologous stem cell transplantation (ASCT) consolidation in responders (4). These approaches have significantly improved progression-free survival (PFS) and overall survival (OS), with some studies reporting median OS exceeding 50 months (3). However, outcomes for relapsed or refractory cases remain poor, with median survival typically less than six months (5).

Accurate treatment response assessment is critical in PCNSL. Gadolinium-enhanced magnetic resonance imaging (MRI) remains the reference standard for monitoring contrast enhancing tumour burden (6). The International Primary CNS Lymphoma Collaborative Group (IPCG) criteria are widely used to describe radiological response, categorising patients into complete response (CR), complete response unconfirmed (CRu), partial response (PR), stable disease (SD), or progressive disease (PD) (6). However, these criteria rely on visual estimates of lesion size and enhancement with high interobserver variability and limited reproducibility (7). Several studies have also questioned the correlation between IPCG-defined response categories and long-term PFS and OS. More objective imaging biomarkers may improve response assessment and prognostication. Quantitative imaging biomarkers may offer greater reproducibility. T1 contrast-enhanced (T1CE) volumetry may better quantify tumour burden and identify subtle interval changes (8, 9). Apparent Diffusion Coefficient (ADC) values from diffusion-weighted imaging (DWI) reflect tumour cellularity, with lower values characteristically associated with PCNSL (10–12). Both T1CE volumetry and ADC have been proposed as biomarkers of IPCG categorical treatment response, but small sample sizes and treatment heterogeneity limit generalisability (for example n=38 (8)). This study investigated baseline and early-treatment MRI in a homogenously treated MATRix cohort. We evaluated T1CE volumetry and ADC metrics as predictors of overall survival and PCNSL-related death.

## Methods

### Patient cohort

Approval for a retrospective analysis of pseudonymised imaging data was granted by the UK Health Research Authority (IRAS ID 326758) with informed consent waived.

Consecutive cases of newly diagnosed PCNSL treated with MATRix were identified from a single-centre PCNSL regional database. Inclusion criteria were: (i) age ≥18 years; (ii) histologically confirmed primary DLBCL of the CNS; (iii) intention-to-treat with MATRix chemotherapy and (iv) pre-treatment MRI including post contrast sequences. Exclusion criteria included unavailable or non-diagnostic imaging. While all patients were treated with an intention-to-treat MATRix regimen, there was some variation in the number of cycles and use of consolidation with ASCT. These reflect real-world outcomes in an ASCT-eligible population, and were not restricted to avoid introducing selection or immortal time bias.

Patients with HIV or other known causes of immunodeficiency were not included, as MATRix is not used in this subgroup at our institution.

### MRI acquisition

MR images were obtained from scanners across centres within the UK East Midlands Radiology Consortium (EMRAD), which includes institutions using 1.5T and 3T systems from multiple vendors. All patients had imaging performed prior to MATRix initiation and after initial treatment. Standardised imaging sequences included axial T2-weighted, FLAIR, pre- and post-gadolinium T1-weighted, and diffusion-weighted imaging (DWI) with apparent diffusion coefficient (ADC) maps generated from at least two b-values (b=0 and b=1000 s/mm²). Sufficient inter-scanner ADC reproducibility is supported by previous validation studies (13).

### T1CE tumour segmentation

**Figure 1:**
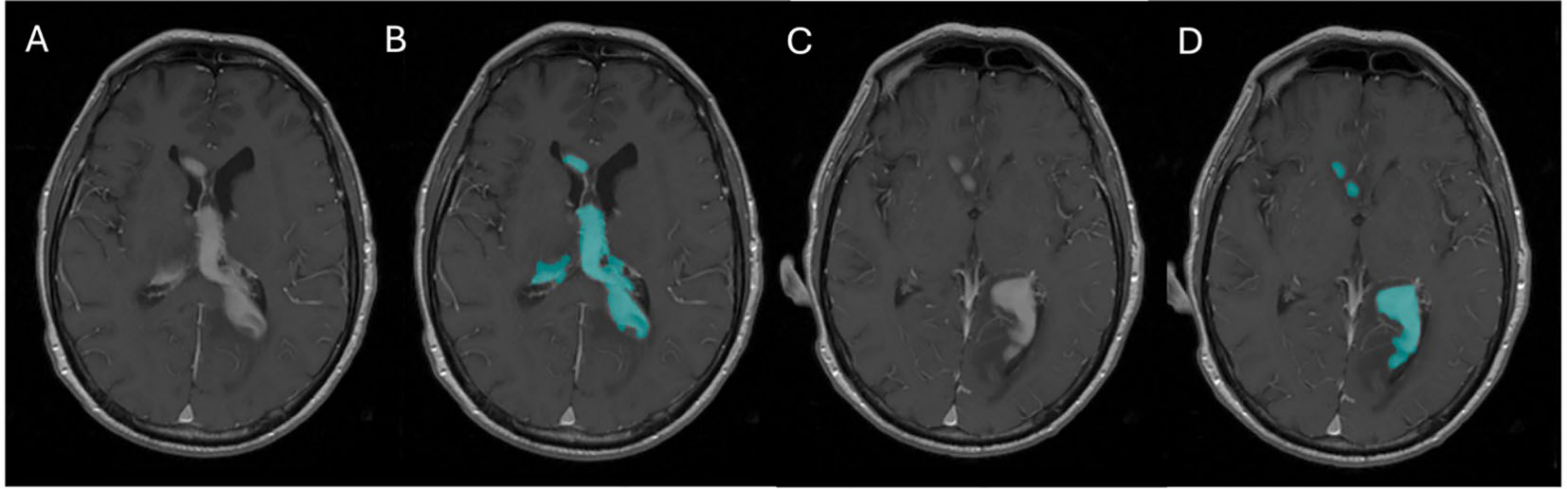
Example of manual segmentation using ITK-SNAP software. Post-contrast axial T1-weighted MR images from a single patient with intra- and periventricular PCNSL, at two cross-sectional levels **(A, C)**. Corresponding segmentations **(B, D)** display cyan overlays indicating manually segmented enhancing tumour volumes used for volumetric analysis.

T1CE tumour volumes were manually segmented using ITK-SNAP software (www.itksnap.org) (14). Segmentations were initially performed by a specifically trained medical student, and in all cases verified by a board-certified neuroradiologist (ST) with 12 years’ experience in neuro-oncology imaging research, blinded to clinical outcomes. For volumetric analysis, the MRI scan immediately preceding MATRix initiation was used; this was typically a scan following diagnostic biopsy, though a minority of patients underwent surgical resections. Tumours were segmented immediately preceding chemotherapy start (baseline) and after completion of MATRix (first response). **Figure 1** illustrates an example segmentation in a patient with intra- and periventricular disease.

The manual segmentation was confined to enhancing regions on post-contrast T1-weighted images, excluding any areas of haemorrhage, necrosis, or blood vessels as described by Cornell et al. (8). In patients with a complete surgical resection, tumour volume was recorded as zero.

### ADC measurements

**Figure 2:**
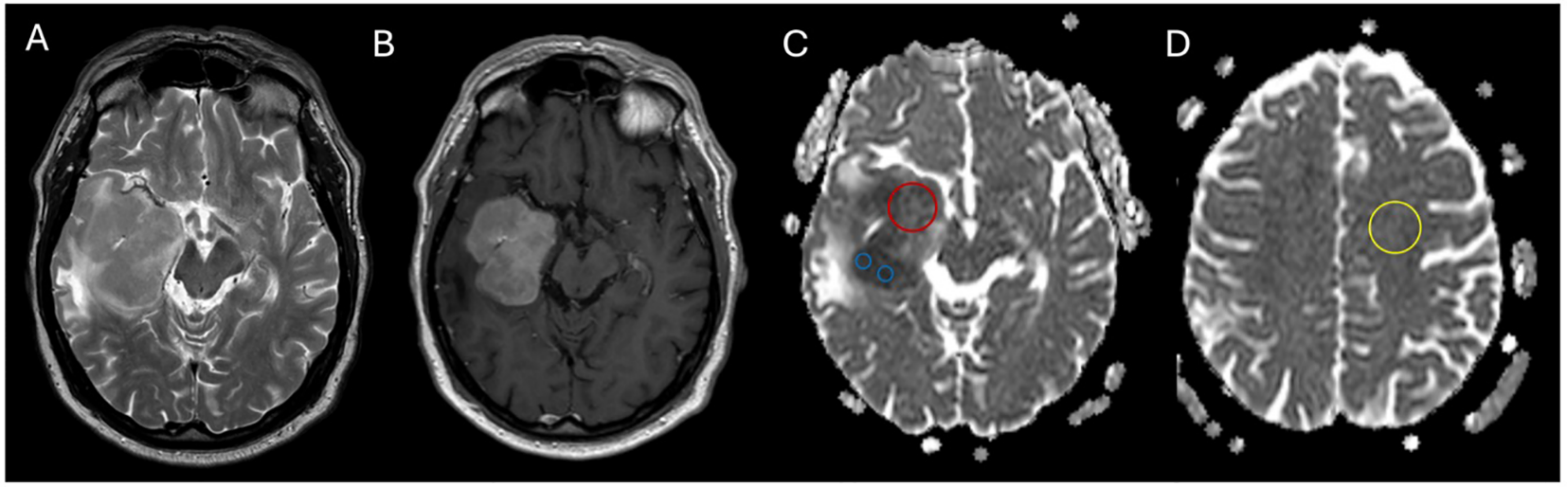
Region-of-interest (ROI) placement for apparent diffusion coefficient (ADC) analysis. Axial T2-weighted **(A)** and post-contrast T1-weighted **(B)** images show an enhancing tumour in the right temporal lobe. ADC maps **(C, D)** demonstrate ROI placement: a large red ROI for ADCmean and small blue ROIs for ADCmin **(C)**, and a yellow ROI in the contralateral normal-appearing white matter at the level of the centrum semiovale **(D)**. Up to three ADCmin ROIs were placed in areas of greatest diffusion restriction, which were not necessarily within the same slice.

Regional ADC measurements were obtained at the earliest available MRI timepoint, as illustrated in **Figure 2**. All measurements were performed by the same board-certified neuroradiologist (ST). Up to three small (20-40mm^2^) regions of interest (ROI) were placed within the areas of visually perceived lowest ADC within contrast enhancing tumour deposits, viewing both sequences side-by-side. From this, the average was recorded as ADCmin.

Next, one ROI ADCmean was placed in the largest contrast-enhancing tumour section to capture as much tissue as possible while avoiding the tumour margin, in order to minimise partial volume effects as previously described by Thust et al. (15).

To minimise any potential influence from scanner technical differences, a further ROI was placed in the contralateral normal-appearing centrum semiovale white matter (ADCnawm) for normalisation. Using this, relative ADC metrics were calculated as follows: rADCmin = ADCmin/ADCnawm and rADCmean = ADC mean/ADCnawm.

### Statistical analysis

Statistical analyses were performed in SPSS version 29 (IBM). OS was defined as the interval from histological diagnosis to death from any cause or last clinical follow up. PFS was defined as the interval from diagnosis to radiological disease progression or last follow up. In addition to analysing OS and PFS using all-cause mortality, a disease-specific survival (DSS) analysis was performed. Deaths attributed to PCNSL were coded as events, with censoring for non-PCNSL deaths (or deaths of unknown cause) and at last follow-up date.

Univariate Cox proportional hazards regression was used to test associations between imaging variables (T1CE tumour volume, ADCmean, ADCmin, mean tumour ADC, rADCmean and rADCmin) and survival outcomes. A p-value <0.05 was considered statistically significant.

## Results

Sixty five patients treated with MATRix at our institution met the inclusion criteria. Their median age was 62 years (IQR 53 – 67), and 28 patients (43%) were female (**Table 1**). The median ECOG performance status was 1. Median serum albumin was 38 g/L (IQR 34 – 41), and the median IELSG score was 1. Baseline T1CE tumour volume was available in 56 patients (86%), with a median volume of 10–470 mm³ (IQR 3078 – 19 768); in 9 cases contrast-enhanced imaging immediately preceding treatment was not available due to tumour resection (tumour volume = 0 mm^3^). These were excluded from the survival analysis to avoid bias. Diffusion weighted-imaging was available in 57 patients (88%) with a median rADCmean of 1.04 (IQR 0.93 – 1.24) and median rADCmin of 0.90 (IQR 0.80 – 1.08).

**Table 1:** Baseline characteristics.

Characteristic	N	Value
Age, years, median (IQR)	65	62 (53 – 67)
Female sex, n (%)	65	28 (43%)
ECOG performance status, median	65	1
Serum albumin, g/L, median (IQR)	65	38 (34 – 41)
IELSG score, median	65	1
Baseline T1CE tumour volume, mm³, median (IQR)	56	10 470 (3078 – 19 768)
rADCmean, median (IQR)	57	1.04 (0.93 – 1.24)
rADCmin, median (IQR)	57	0.90 (0.80 – 1.08)

At the last follow up, 26 patients had died from any cause, of which 13 deaths were attributable to PCNSL. There were 22 events for the all-cause PFS analysis and 12 events for the DSS analysis.

The median follow-up duration was 39 months (IQR 20–65). The median overall survival (OS) was 58 months (IQR 36–80), and the median progression-free survival (PFS) was 34 months (IQR 12–62).

In univariate Cox regression for all-cause mortality, none of the clinical or imaging variables were associated with overall survival (**Table 2**). Age, albumin, baseline T1CE tumour volume, IELSG score and ECOG performance status were not significantly associated with OS. Tumour volume and percentage volumetric change at first-response assessment were not associated with OS. Moreover, no diffusion-based metric, including ADCmean, ADCmin, rADCmean or rADCmin, demonstrated a significant association.

**Table 2:** Cox regression analysis for all cause mortality (OS) and progression (PFS).

Variable	Events (OS)	HR for OS (p)	Events (PFS)	HR for PFS (p)
Age	26	1.01 (0.53)	22	1.01 (0.66)
Albumin	26	1.02 (0.63)	22	1.02 (0.63)
Baseline tumour volume	22	1.00 (0.43)	22	1.00 (0.27)
IELSG score	26	0.88 (0.48)	22	0.80 (0.28)
ECOG	26	0.87 (0.49)	22	0.83 (0.42)
First response tumour volume	20	1.00 (0.40)	19	1.00 (0.27)
% volume change	19	1.00 (0.76)	19	1.00 (0.55)
ADCmean	21	1.00 (0.52)	20	1.00 (0.69)
rADCmean	20	0.61 (0.46)	19	0.70 (0.55)
ADCmin	21	1.00 (0.87)	20	1.00 (0.93)
rADCmin	20	0.72 (0.64)	19	0.76 (0.68)

Similarly, PFS analyses showed no significant associations between any variable and PFS (**Table 2**). Baseline tumour volume, response volume, percentage tumour reduction and ADC metrics were not predictive of progression.

The disease specific analyses, restricted to deaths attributed to PCNSL, identified similar findings (**Table 3**). None of the clinical variables, including age, albumin, IELSG score or ECOG status, showed significant associations with DSS. Baseline tumour volume, first response volume and percentage volume change were also not associated with disease specific mortality. ADCmean, ADCmin, rADCmean and rADCmin showed no significant relationships with outcomes.

**Table 3:** Cox regression analysis for PCNSL specific mortality (PCNSL-OS) and progression (PCNSL-PFS).

Variable	Events (PCNSL-OS)	HR for PCNSL-OS (p)	Events (PCNSL-PFS)	HR for PCNSL-PFS (p)
Age	13	0.98 (0.40)	12	0.98 (0.42)
Albumin	13	1.11 (0.12)	12	1.09 (0.22)
Baseline tumour volume	12	1.00 (0.74)	12	1.00 (0.74)
IELSG score	13	0.68 (0.18)	12	0.62 (0.13)
ECOG	13	0.87 (0.61)	12	0.65 (0.20)
First response tumour volume	12	1.00 (0.70)	12	1.00 (0.70)
% volume change	12	1.00 (0.96)	12	0.99 (0.38)
ADCmean	10	1.00 (0.24)	10	1.00 (0.29)
rADCmean	9	0.23 (0.34)	9	0.31 (0.42)
ADCmin	10	1.00 (0.72)	10	1.00 (0.72)
rADCmin	9	0.60 (0.67)	9	0.67 (0.72)

No variable was associated with PCNSL-specific PFS. Baseline tumour volume, response volume, percentage change and all diffusion metrics yielded non-significant hazard ratios in the disease specific PFS models.

## Discussion

This study investigated whether baseline quantitative MRI biomarkers, specifically T1CE tumour volume, volume dynamics or ADC derived diffusion metrics are predictive of survival outcomes in newly diagnosed PCNSL treated with MATRix chemotherapy. When applied to real-world patient data, none of the tested imaging variables showed significant associations with overall or progression free survival. Given the rarity of PCNSL, our cohort represents a moderately large dataset, which benefits from homogenous standard of care treatment. Previous PCNSL MRI biomarker studies have typically included smaller study cohorts (for example Barajas et al. n=18, Zacharia et al. n=20 and Chien et al. n=52) with more treatment heterogeneity (10, 11, 16). The negative study findings in a more uniformly treated cohort highlight the complexity in predicting PCNSL prognosis by both clinical and imaging parameters. Two principal motivations for identifying prognostic biomarkers pre-treatment include the substantial toxicities associated with the MATRix regimen, alongside the risk of early disease progression (2). Reliable baseline risk stratification could support a concept of treatment de-escalation for patients with good risk PCNSL or conversely individuals or early intensification for high-risk patients, while also improving certainty during patient counselling. The homogeneous use of MATRix strengthens interpretability when compared with older studies featuring mixed radiotherapy and non-methotrexate regimens with potential impact on biomarker interpretation (3). Nevertheless, our limited sample size still raises the possibility of a type II error.

Prior work on tumour burden in PCNSL has shown inconsistent prognostic associations. Lauer et al. reported that lower baseline volumes and a large early volumetric reduction predicted improved survival for a slightly larger cohort (n=93 PCNSL) (9), whereas Huntoon et al. found no relationship between tumour size and outcome (n=79 PCNSL) (17). Systemic lymphoma literature strongly supports tumour burden as a prognostic factor, including in diffuse large B cell lymphoma where high metabolic tumour volume independently predicted poorer survival (n=301) (18). However, this relationship does not appear universal; studies of extranodal or CNS involved systemic lymphomas have reported weaker or absent associations between bulk disease and outcome (19). These discrepancies indicate that tumour volume may not be the principal determinant of PNCSL outcome. Specifically, lymphoma growth and relapse risk may be shaped by systemic and local neuroimmune microenvironment factors. For example, multiple studies have shown that systemic inflammatory indices, such as neutrophil-to-lymphocyte ratio and lymphocyte-to-monocyte ratio, stratify survival in PCNSL independently of tumour burden (20, 21). Moreover, a study which performed whole-genome profiling of 68 cases of PCNSL showed that high copy number variation and recurrent mutations, particularly CD79B and TMSB4X, independently predicted outcome leading to proposals for gene-based prognostic models (22).

Diffusion metrics have been proposed as predictive of PCNSL burden due to the characteristic of low ADC as a correlate for packed malignant lymphoid cells on microscopy (8, 12). However, it is unclear to what extent ADC informs on disease severity and/or prognosis. In glioma, Maynard et al. demonstrated that ADC ROI measurements show excellent reproducibility and are useful in identifying biologically aggressive disease (23). Their role in PCNSL is less clear. Several groups reported prognostic associations between ADC and outcome, including Baek et al. (n=67) and Chien et al. (n=52), who linked lower ADC with poorer survival or refractory disease (12, 16). Cornell et al. observed baseline ADCmean differed across MATRix IPCG response categories (8). Despite a partial overlap in our patient cohort, the current study did not consolidate these associations. Importantly, ADC is influenced by cellularity but may not directly correlate with disease spatial extent, tumour biology, nor systemic immune competence or local vulnerability of brain microenvironments. Immune permissive factors such as HIV infection, chronic immunosuppression and age related immunosenescence are well recognised contributors to PCNSL pathogenesis (1), none of which are fully captured by diffusion parameters.

There is limited evidence that advanced MRI techniques, including dynamic susceptibility contrast (DSC) and dynamic contrast-enhanced (DCE) perfusion and diffusion tensor imaging, can provide additional biological information beyond standard MRI sequences (24). Such measures could theoretically enhance prognostic modelling in PCNSL. However, the incorporation of these techniques into routine PCNSL practice remains negligible, potentially impacted by multiple factors including clinical pressures on scanner time or uncertainties regarding interpretation (25). These practical constraints highlight the need for prognostic tools that remain robust within standard clinical MRI workflows.

Taken together, the study results align to emerging evidence that prognosis in PCNSL is driven by multiple independent biological domains not fully captured by imaging. Computational approaches and systemic inflammatory markers have recently demonstrated stronger prognostic signal than baseline MRI alone (9, 21), encouraging the development of risk models incorporate molecular, pathological and systemic factors. In this study, radiomics analysis was not attempted due to the small cohort size, which could risk model overfitting. Instead, the remit was to investigate ‘real world’ biomarkers that could be applied without the need for specialist computation.

While baseline MRI remains essential for diagnosis, our research suggests it may have limited standalone prognostic value. We acknowledge that the cohort size, missing cause of death data and follow-up limitations may constrain generalisability. An additional limitation relates to the timing of baseline MRI acquisition. Post−operative imaging and peri−operative corticosteroid use may have influenced contrast enhancement and tumour volume measurements in a subset of cases. Although this approach reflects real−world clinical practice, it may have introduced variability in baseline tumour burden estimation and potentially attenuated prognostic associations. We also acknowledge that a degree of patient overlap with a previous two-centre study by Cornell et al. is possible (8). Approximately 20 patients from our centre were included in that study, although is not practically verifiable owing to data anonymisation protocols which we recognise this as another limitation. These results should be interpreted cautiously but contribute valuable negative evidence to the literature, underscoring the need for integrated, multimodal prognostic strategies in PCNSL.

## Conclusions

Neither contrast enhancing tumour volume nor ADC based diffusion metrics may reliably predict survival in MATRix-treated PCNSL. Our research findings add important negative evidence to a literature dominated by small heterogeneous datasets with a risk of positive publication bias. The results also highlight the need for validation of any proposed biomarkers across real world cohorts. Future work should prioritise multimodal prognostic frameworks that integrate imaging with molecular and immune based markers to achieve accurate prognostic stratification in PCNSL.

## Data Availability

The raw data supporting the conclusions of this article will be made available by the authors, without undue reservation.
